# Targeting Zika Virus with New Brain- and Placenta-Crossing Peptide–Porphyrin Conjugates

**DOI:** 10.3390/pharmaceutics14040738

**Published:** 2022-03-29

**Authors:** Toni Todorovski, Diogo A. Mendonça, Lorena O. Fernandes-Siqueira, Christine Cruz-Oliveira, Giuseppina Guida, Javier Valle, Marco Cavaco, Fernanda I. V. Limas, Vera Neves, Íris Cadima-Couto, Sira Defaus, Ana Salomé Veiga, Andrea T. Da Poian, Miguel A. R. B. Castanho, David Andreu

**Affiliations:** 1 Department of Medicine and Life Sciences, Universitat Pompeu Fabra, 08003 Barcelona, Spain; toni.todorovski@upf.edu (T.T.); giuseppina.guida@upf.edu (G.G.); javier.valle@upf.edu (J.V.); sira.defaus@upf.edu (S.D.); 2 Instituto de Medicina Molecular, Faculdade de Medicina, Universidade de Lisboa, 1649-028 Lisbon, Portugal; diogo.mendonca@medicina.ulisboa.pt (D.A.M.); christine.oliveira@medicina.ulisboa.pt (C.C.-O.); mcavaco@medicina.ulisboa.pt (M.C.); veraneves@medicina.ulisboa.pt (V.N.); cicouto@medicina.ulisboa.pt (Í.C.-C.); aveiga@medicina.ulisboa.pt (A.S.V.); 3 Instituto de Bioquímica Médica Leopoldo de Meis, Universidade Federal do Rio de Janeiro, Rio de Janeiro 21941-902, Brazil; losiqueira@bioqmed.ufrj.br (L.O.F.-S.); fernanda.ignaciovl@gmail.com (F.I.V.L.)

**Keywords:** peptide-drug conjugates, blood–brain barrier, blood–placental barrier, Zika virus, BBB shuttles, porphyrins, antivirals

## Abstract

Viral disease outbreaks affect hundreds of millions of people worldwide and remain a serious threat to global health. The current SARS-CoV-2 pandemic and other recent geographically- confined viral outbreaks (severe acute respiratory syndrome (SARS), Ebola, dengue, zika and ever-recurring seasonal influenza), also with devastating tolls at sanitary and socio-economic levels, are sobering reminders in this respect. Among the respective pathogenic agents, Zika virus (ZIKV), transmitted by *Aedes* mosquito vectors and causing the eponymous fever, is particularly insidious in that infection during pregnancy results in complications such as foetal loss, preterm birth or irreversible brain abnormalities, including microcephaly. So far, there is no effective remedy for ZIKV infection, mainly due to the limited ability of antiviral drugs to cross blood–placental and/or blood–brain barriers (BPB and BBB, respectively). Despite its restricted permeability, the BBB is penetrable by a variety of molecules, mainly peptide-based, and named BBB peptide shuttles (BBBpS), able to ferry various payloads (e.g., drugs, antibodies, etc.) into the brain. Recently, we have described peptide–porphyrin conjugates (PPCs) as successful BBBpS-associated drug leads for HIV, an enveloped virus in which group ZIKV also belongs. Herein, we report on several brain-directed, low-toxicity PPCs capable of targeting ZIKV. One of the conjugates, PP-P1, crossing both BPB and BBB, has shown to be effective against ZIKV (IC_50_ 1.08 µM) and has high serum stability (t_1/2_ ca. 22 h) without altering cell viability at all tested concentrations. Peptide–porphyrin conjugation stands out as a promising strategy to fill the ZIKV treatment gap.

## 1. Introduction

Among human-targeting viruses, brain-penetrating ones such as Zika virus (ZIKV), Dengue virus, or HIV pose formidable hurdles to pathogen targeting, ZIKV being a particularly illustrative case in this regard.

ZIKV, a member of the *Flaviviridae* family, [1] causes the namesake zika fever, a zoonosis transmitted by *Aedes* mosquito bites [2,3]. The direst consequences of ZIKV infection concern pregnant women, as virus transmission to the foetus causes irreversible congenital brain abnormalities, including microcephaly [4]—a predicament involving over 1400 cases during the 2016 outbreak in Brazil [5]. ZIKV infection can also result in further complications such as foetal loss, preterm birth or other birth defects, and can also trigger Guillain–Barré syndrome [4], encephalomyelitis [6], and similar conditions in adults. ZIKV was detected in a microcephalic foetus ca. 32 weeks after maternal exposure, suggesting long persistence in the foetal brain [3].

The World Health Organization (WHO) has classified ZIKV amongst the viruses posing the greatest public health risk due to its epidemic potential. WHO recommendations have focused on the development of inactive and/or other “non-live” vaccines [7], with a number of vaccine candidates showing some promise in human clinical trials [8]. An alternative approach, involving genetic modification of the *Aedes* mosquito transmission vector, has also raised attention, though conclusive data on its effectiveness is lacking [9]. In more conventional therapeutic approaches, any anti-ZIKV drug development effort must face the challenge of moving the drug across the blood–placental (BPB) and blood–brain (BBB) barriers to reach ZIKV at its most sensitive sites of action: the brain or the foetus [10,11]. The BBB is a natural protective barrier made up by endothelial cells (ECs), astrocytes and pericytes, whose unique composition allows tight regulation of central nervous system (CNS) homeostasis, which is critical for proper neuronal function, as well as for protection from toxins, pathogens, inflammation, injury, and disease. BBB fulfils this role by having very selective transporters for a limited number of specific molecular entities circulating in the blood. For its part, the BPB, consisting of trophoblastic epithelium, is a “leakier” barrier, blocking mainly the diffusion of large molecules.

In the last two decades, a variety of molecules, broadly described as BBB shuttles, have been shown to successfully overcome BBB permeability [12,13,14,15]. The “BBB shuttle” term was first introduced by Pardridge [16] to denote the ability of such agents, chemically modified or not, to ferry brain-targeting drug payloads into and out of the brain through the BBB. As many such shuttles are peptide-based, the term “BBB peptide shuttle” [BBBpS] has been more recently coined [17].

Strategies for conjugating BBBpS to drug payloads have been actively explored over recent decades, with the number of new shuttles steadily increasing [12,13,14,15,18,19,20]. Some recent entries, moreover, have shown to be capable not only of carrying drugs into but also removing toxins from the brain, preventing their accumulation [21,22]. For our part, we have reported that peptide–porphyrin conjugates (PPCs), where a BBBpS and an antiviral porphyrin are covalently linked by an amide bond (amino and carboxyl groups in BBBpS and porphyrin, respectively) can successfully pass the BBB and act against brain-targeting viruses such as HIV [23]. As for the BPB, the literature is scarce, with only a few described examples of peptides able to pass it [24]. 

**Scheme 1:**
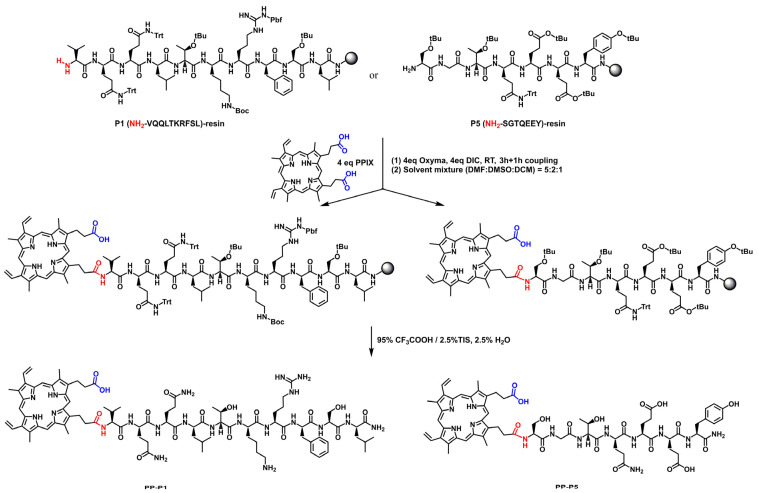
On-resin conjugation strategy at the N-terminus of a BBBpS.

Herein, we report our results in developing new PPCs able to penetrate both BPB and BBB and act against ZIKV. The PPC production strategy, involving porphyrin (P), *C*- or *N*-terminal conjugation to a BBBpS, has been detailed in a recent publication and is illustrated in Scheme 1 and Scheme S1 [23]. In this paper we describe eight PPCs (Table 1) resulting from the combination of four BBBpS (Table S1) and two porphyrins, and their evaluation in terms of barrier crossing and anti-ZIKV activity. One of the conjugates, PP-P1, emerges as particularly effective against ZIKV, having also the ability to translocate across BPB and BBB.

## 2. Materials and Methods

### 2.1. Chemicals and Reagents

Mesoporphyrin IX dihydrochloride and protoporphyrin IX were from Frontier Scientific, Inc (Logan, UT, USA). HPLC-grade DMF, DCM and MeCN were from Fisher (Madrid, Spain), and NMP was from Sigma-Aldrich (Madrid, Spain). Fmoc-amino acids, DIC and Oxyma were from Iris Biotech (Marktredwitz, Germany). 

### 2.2. Cells and Cell Culture Reagents

HBEC-5i human brain endothelia (ATCC-CRL-3245) and JEG-3 placental trophoblast (ATCC-HTB-36) cell lines, and Eagle’s Minimal Essential Medium (EMEM) were from ATCC (Manassas, VA, USA). Dulbecco’s Modified Eagle Medium: Nutrient Mixture F-12 (DMEM:F12), foetal bovine serum (FBS), penicillin-streptomycin (Pen-Strep), attachment factor protein and trypsin-EDTA were from Gibco (Thermo-Fisher, Waltham, MA, USA). AlamarBlue^®^ reagent was from Invitrogen (Thermo-Fisher). Endothelial cell growth supplement from bovine neural tissue (ECGS) was from Sigma-Aldrich (Merck, Darmstadt, Germany). Penicillin and streptomycin were from LGC Biotecnologia (Cotia, SP, Brazil). 

### 2.3. Peptide Synthesis

P1, P2, P5 and P6 were made by Fmoc solid phase synthesis on a Liberty Blue (CEM Matthews, NC, USA) instrument using Protide resin (0.54 mmol/g) at 0.1 mmol scale. The P2 and P6 sequences are variants of P1 and P5 (Table S1), respectively, elongated at their *C*-termini with an extra Lys residue, orthogonally protected with the monomethoxytrityl (Mmt) group. Other side chain protecting groups were *tert*-butyl (Ser, Thr, Glu), trityl (Gln), Boc (Lys) and 2,2,4,6,7-pentamethyldihydrobenzofuran-5-sulfonyl (Arg). The identity of the resin-bound peptides prior to porphyrin conjugation was assessed by resin test cleavage (RTC) combined with LC-MS analysis. Briefly, ca. 2 mg of peptide resin were reacted with 170 µL of TFA-TIS-H_2_O (95:2.5:2.5, *v*/*v*/*v*) for 90 min at r.t. Then, 1 mL of cold diethyl ether was added, and the suspension was centrifuged at 12,400 rpm for 8 min. The supernatant was removed and the pellet, after drying, was dissolved in 15% MeCN/0.1% TFA (P1 and P2) or 0.1 % TEA (P5 and P6) and analysed by LC-MS (see below).

### 2.4. Conjugation Chemistry 

Reactions were performed at 50 µM scale (1 eq in all further calculations) as reported [23]. Briefly, 4 eq of porphyrin (MPIX or PPIX), mixed with 4 eq oxyma, were dissolved, respectively, in NMP:DCM:DMF (3:2:1, *v*/*v*/*v*) or DMF:DMSO:DCM (5:2:1, *v*/*v*/*v*), at a final 0.05 M concentration. The solution was mixed with 4 eq DIC (plus 8 eq DIPEA in the case of MPIX) and added to the peptide resin. In *C*-terminal conjugations (P2 and P6), prior to porphyrin coupling, the Mmt group at the *C*-terminal Lys side chain was selectively removed by 1% TFA in DCM (5 × 1 min) followed by DCM washes: the cycle was repeated until no more yellow colour (presence of Mmt) was observed. After 3 h at r.t., conjugation with fresh reagents was repeated for an additional 1 h. A slightly modified RTC on a small aliquot of the PPC-resin (cleavage as above), with N_2_ flush evaporation instead of ether precipitation and the residue dissolved in 20% MeCN for LC-MS analysis, was used to confirm conjugate identity. Next, the bulk PPC-resin was likewise cleaved, the resulting solution was N_2_ flush-evaporated, dissolved in H_2_O/MeCN (80:20 *v*/*v*) and lyophilized.

### 2.5. PPC Purification

Conjugates were purified by semi-preparative HPLC on an LC20-AP instrument (Shimadzu, Kyoto, Japan) using a Gemini C_18_ column (10 µm, 110 Å, 10 × 250 mm, Phenomenex, Torrance, CA, USA). Each conjugate was dissolved in 22% MeCN/25% DMF/H_2_O and a linear 15-95% MeCN gradient in H_2_O (0.1 % TFA) over 40 min at 6 mL/min flow rate was applied. The fractions were analysed by LC-MS and those with >90% homogeneity were collected, combined, lyophilized and stored at −20 °C.

### 2.6. LC-MS Analysis

Crude peptides or conjugates after RTC were dissolved in 15% or 20% MeCN, respectively, and analysed on a LCMS-2010 EV instrument (Shimadzu). For the analysis, 15 µL of a ~1 mg/mL solution were injected on an Aeris XB-C_18_ column (3.6 µm, 150 × 4.6 mm, Phenomenex) eluted with a linear 5–95% MeCN gradient into 0.1 % FA in H_2_O over 15 min at 1 mL/min flow rate. MS detection was set to 200–2000 *m/z*. Purified conjugates were dissolved at 1 mg/mL in 20% MeCN and analysed by LC-MS using a linear 10–60% MeCN gradient into 0.1% FA in H_2_O over 15 min, other parameters as above.

### 2.7. In Vitro BBB Translocation Assay

HBEC-5i cells were cultured as a monolayer on T-flasks in DMEM:F12 supplemented with 10% (*v*/*v*) FBS, 1% (*v*/*v*) Pen-Strep and 1% (*v*/*v*) ECGS. Cells were grown in a humidified atmosphere of 5% CO_2_ at 37 °C (MCO-18AIC (UV), Sanyo, Japan) with the medium changed every other day. Cells were allowed to grow until confluence in a culture T-flask, then carefully harvested with trypsin-EDTA and seeded (8000 cells/well) into tissue culture 24-well inserts (transparent polyester membrane with 1.0 µm pores) (BD Falcon-Corning, Corning, NY, USA) pre-coated with attachment factor protein solution. The medium was changed every other day for 5–8 days, afterwards cells were washed with 1 × PBS, followed by DMEM:F12 medium without phenol red. Next, PPCs diluted in DMEM:F12 without phenol red, at a final 10 µM concentration, were added to the apical side of the in vitro BBB model. Experiments were performed on different days using independently grown cell cultures.

PPC translocation was determined by fluorescence intensity (λ_ex_ = 410 nm, λ_em_ = 625 nm for MP conjugates: λ_ex_ = 410 nm, λ_em_ = 635 nm for PP conjugates). P1-carboxyfluorescein (CF-P1) and P5-carboxyfluorescein (CF-P5) were used as positive translocation controls (λ_ex_ = 492 nm and λ_em_ = 517 nm). After 24 h incubation, samples from the basolateral side were collected and analysed. Fluorescence was measured in a Varioskan Lux plate reader (Thermo Scientific). PPC translocation (%) was calculated as follows:

(1) $$\mathrm{PPC}\;\mathrm{translocation}\;\left(\%\right)=\frac{F_{i}-Fcells}{F_{PPC}-F_{Medium}}\times 100$$

where *F_i_* is the fluorescence intensity of the sample collected at the basolateral side, *F_cells_* is the intrinsic fluorescence intensity of cells without *PPC* incubation, *F_PPC_* is the intensity of total *PPC* initially added to the transwell apical side, and *F_Medium_* is the intensity of DMEM:F12 medium without phenol red. Three independent replicates were performed.

### 2.8. In Vitro BBB Integrity Assay

After 24 h incubation with PPCs, cells were washed with PBS and DMEM:F12 medium without phenol red. Then, 4 kDa fluorescein isothiocyanate-dextran (FD4) (Sigma-Aldrich, Madrid, Spain) in DMEM:F12 without phenol red, previously diluted to an absorbance of 0.1, was added to the apical side and incubated for 2 h. Samples were collected at the basolateral side, and fluorescence intensity was measured at λ_ex_ = 493 nm and λ_em_ = 520 nm. Barrier integrity was evaluated from FD4 permeability as follows:

(2) $$FD4\;\mathrm{Permeability}\;\left(\%\right)=\frac{F_{i}-F_{cells}}{F_{FD4}-F_{Medium}}\times 100$$

where *F_i_* is the fluorescence intensity of the sample at the basolateral side, *F_cells_* is the intrinsic fluorescence intensity of cells without FD4 incubation, *F_FD4_* is the intensity of FD4 stock initially added to the apical side, and *F_Medium_* is the intensity of DMEM:F12 medium without phenol red. Three independent replicates were performed.

### 2.9. In Vitro BPB Translocation and Integrity Assay

JEG-3 cells were cultured as a monolayer on T-flasks in EMEM supplemented with 10% (*v*/*v*) FBS and 1% (*v*/*v*) Pen-Strep. Cells were grown in a humidified atmosphere of 5% CO_2_ at 37 °C with the medium changed every other day. Cells were allowed to grow until confluence in a culture T-flask, then carefully harvested with trypsin-EDTA and seeded (3000 cells/well) onto rat-tail collagen-coated tissue culture 24-well inserts. A cellular monolayer with low permeability was formed 5 days after the incubation on the tissue culture insert. BPB translocation and integrity measurement methods are identical to those for the BBB in vitro model. Three independent replicates were performed.

### 2.10. Virus Samples

ZIKV^BR^ was isolated from a febrile case in the state of Pernambuco, Brazil (gene bank ref. number KX197192) and was kindly provided by Dr. Ernesto T. A Marques Jr (Centro de Pesquisas Aggeu Magalhães, Fiocruz, Pernambuco, Brazil). ZIKV^BR^ was propagated in C6/36 cells using a multiplicity of infection (MOI) of 0.01. After infection, cells were cultured for 7 days. The culture medium collected from the infected cultures was centrifuged at 700× *g* to remove cellular debris, stored in aliquots at −80 °C and titrated by plaque assay as described elsewhere [25].

### 2.11. ZIKV Inactivation Assay

To determine the effect of PPCs on ZIKV^BR^, 10^5^ plaque forming units (PFU) were pre-treated with different PPC concentrations in the 0.01–50 µM range for 1 h at 37 °C, in the dark. After treatment, viral samples were serially diluted (10-fold) for titration [25]. Samples were incubated for 1 h at 37 °C and 5% CO_2_ with Vero cells. After infection, the medium was removed and 1 mL DMEM containing 1% (*v*/*v*) FBS, 100 U/mL penicillin, 100 µg/mL streptomycin and 1.5% carboxymethylcellulose was added to each well. The plates were incubated at 37 °C and 5% CO_2_. After 5 days, cells were fixed by 1 mL of 4% (*v*/*v*) formaldehyde in H_2_O for 30 min. Each plate was washed and stained with a 1% (*v*/*v*) crystal violet, 20% (*v*/*v*) ethanol solution. The number of plaques on each well was counted and corrected for well dilution to determine the pfu/mL number. The half-maximum inhibitory concentration (IC_50_) was determined by nonlinear regression with sigmoidal profile and variable slope using the software Graphpad Prism (version 6.0, Graphpad Software, San Diego, CA, USA), only on treatments that achieved total inhibition. Three independent replicates were performed.

### 2.12. Cell Viability Assay

HBEC-5i cells and JEG-3 cells were plated in attachment factor protein and rat-tail collagen, respectively, pre-coated 96-well flat bottom clear, black polystyrene plate (Corning, New York, NY, USA) as previously described [23]. Afterwards cells were cultured in complete medium at 37 °C in a 5% CO_2_ atmosphere, with medium replaced every 2 days. When the cellular monolayer was formed, cells were treated with various PPC concentrations in the 6.25–50 µM range, for 24 h at 37 °C in a 5% CO_2_ atmosphere. Viability was evaluated by the CellTiter-Blue^®^ assay (Promega, Madison, WI, USA), based on resazurin reduction into highly fluorescent resorufin by metabolically active cells. By distinguishing metabolic from non-metabolic cells, cytotoxicity can be indirectly determined. After incubation, cells were washed with PBS, pH 7.4, and 15 µL of CellTiterBlue^®^ reagent in 100 µL of complete medium was added to the cells and incubated for 1.5 h at 37 °C in 5% CO_2_. Fluorescence (λ_ex_ = 560 nm, λ_em_ = 590 nm), was measured in a Varioskan Lux plate reader. Complete medium and medium containing 0.25% Triton X-100 were used as positive and negative controls (100 and 0% viability), respectively. Cell viability (%) was determined as:

(3) $$\mathrm{Cell}\;\mathrm{viability}\;\left(\%\right)=\frac{F_{treated}-F_{blank}}{F_{non\;treated}-F_{blank}}\times 100$$

where *F_treated_* is the fluorescence intensity of PPC-treated cells, *F_non treated_* is the fluorescence of untreated cells and *F_blank_* is the fluorescence of CellTiterBlue^®^ reagent in complete medium without cells. Three independent replicates were performed.

### 2.13. Serum Stability

Conjugates, peptides P1, P2 and P5 were dissolved at 0.1 mM in HEPES buffer (10 mM, NaCl 150 mM, pH 7.8), with 1.4 % DMSO added for MP-P1 and P2-MP to improve solubility. Next, 600 µL of conjugate/peptide solution were mixed (1:1 *v*/*v*) with 600 µL of human serum, to a 0.05 mM final concentration. The mixture was incubated at 37 °C and, at various time points, 50 µL (in triplicate) were taken and precipitated with 200 µL cold methanol. The samples were centrifuged at 13,000 rpm, 10 min, +4 °C. Afterwards, 50 µL of each supernatant were injected on an LC-20AD instrument (Shimadzu) equipped with a Luna C18 column (4.6 mm × 50 mm, 3 µm, Phenomenex, Torrance, CA, USA) and analysed using a 0–95% linear gradient of MeCN into 0.1% TFA in H_2_O over 15 min with 1 mL/min flow rate. PDA detection at 220 nm and 401 nm was used and the half-life times were calculated by peak area integration at 401 nm, the λ_max_ of MP and PP. Controls included blank serum (25 µL each of 1:1 *v*/*v* serum + HEPES buffer, then precipitation with 200 µL cold methanol), untreated conjugate/peptide (25 µL of 0.1 mM stock in HEPES buffer + 25 µL HEPES buffer, then precipitation with 200 µL cold methanol), and a zero-time sample (200 µL cold methanol + 25 µL serum + 25 µL 0.1 mM conjugate/peptide stock); the conjugate/peptide was added last to avoid any contact with protease. Controls were analysed by LC and LC-MS as above. Three independent replicates were performed.

## 3. Results and Discussion

### 3.1. BBBpS—Porphyrin Conjugate Design

Peptide–porphyrin conjugates (PPCs) are regarded as promising leads against viral and bacterial infections [26,27,28,29,30]. The antiviral properties of porphyrins make them attractive for treating viral diseases, including those caused by enveloped viruses such as HIV, ZIKV, and DENV [25,31,32], while BBBpS conjugation allows to overcome obstacles such as porphyrin poor water solubility and low cellular uptake. We have recently shown that PPCs involving a covalent (amide) link between amino (*N*-terminal or Lys side chain) groups of a BBBpS and porphyrin carboxyl groups can successfully pass the BBB and act against HIV without significant cytotoxic activity [23]. For HIV, the most plausible inactivation mechanism seems to be envelope targeting with severe perturbation of lipid bilayer integrity. Since ZIKV is also enveloped, we decided to exploit the same approach by means of a PPC library with potential BPB and BBB-crossing abilities. Specifically, the chemistry in Scheme 1 and Scheme S1 [23] has been applied to two different BBBpS conjugation sites (*N*- or *C*-terminal) and two different porphyrins (MP and PP). All the eight conjugates in Table 1 were obtained at >90% purity and in 10–30% final yields after HPLC purification (Table S3).

### 3.2. In Vitro BBB and BPB Translocation Assays

**Figure 1:**
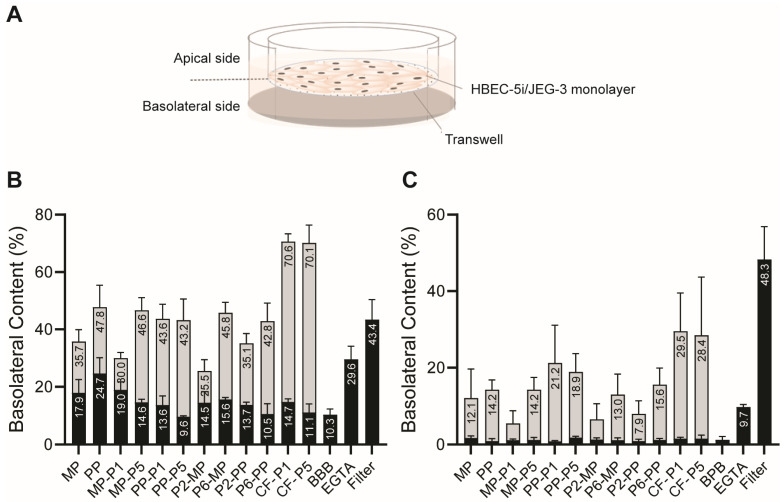
In vitro PPC translocation. (**A**) Schematic representation of the BBB and BPB model transwell system. (**B**,**C**) in vitro BBB and BPB translocation measurements. PPC translocation values are depicted in grey, while FD4 permeability values post-translocation are in black (BBB and BPB—only cells, EGTA—tight junctions disruption control, Filter—no cells).

At variance with the previous studies [23], where a mouse BBB in vitro model was used, here we used a human model, a cellular barrier formed by human endothelial brain cells (HBEC-5i) [33] grown in a cell culture insert, allowing formation of a cell monolayer with restrictive paracellular permeability and expression of essential BBB transporters. Placed into a transwell system, the cellular monolayer divides two chambers mimicking the blood and brain sides (Figure 1A) and allowing quantification of drug translocation. In this device, all PPCs efficiently translocate the monolayer, five of them—MP-P5, PP-P1, PP-P5, P6-MP and P6-PP—reaching values above 43% (Figure 1B). Although lower than those of non-conjugated peptides P1 and P5, the trans-BBB scores for these PPCs are in the range of best-performing leads reported in peptide-drug antiretroviral and anti-Alzheimer therapeutic approaches [34,35,36]. Conjugation improves translocation for MP bound to BBBpS P5 or P6 relative to free MP (Figure 1B). Moreover, free PP significantly compromises the integrity of the barrier, as shown by the 2.5-fold increase in FD4 permeability over control values (24.7 and 10.3%, respectively), approaching EGTA disruption values, a behaviour not observed with the conjugates. 

To evaluate in vitro BPB translocation, the above-described transwell system was modified to mimic a human placental barrier, using trophoblast cultures (JEG-3 cell line) [37] and optimized to ensure cell monolayers with low permeability (Figure S1). In this setup, the same five PPCs shown to better translocate the BBB in vitro were also those with higher trans-BPB capabilities (Figure 1C), although scores were significantly lower than for BBB translocation. It seems, thus, reasonable to assume that translocation mechanisms for PPCs differ between the two barriers. It is worth mentioning at this point that, while adsorptive-mediated transcytosis (AMT) is deemed the preferential BBB translocation mechanism by our BBBpS [21,22], and the most likely mechanism for the PPCs in this study, it has not yet been reported as a BPB transmigration mechanism [38]. Passive diffusion, the preferential transmigration mechanism used by trans-BPB compounds [39], seems to be non-favoured by PPCs, given the disparity in translocation values between the PPCs and the antipyrine positive control (Figure S1C). At any rate, five PPCs with significant BBB and BPB translocation abilities suitable for ZIKV inactivation were identified. It is also worth noting that none of the PPCs altered cell viability (HBEC-5i, JEG-3) at concentrations up to 50 µM (data not shown). 

### 3.3. ZIKV Inactivation

The five PPCs that successfully translocated BBB and BPB were evaluated for ZIKV inactivation in vitro, using a plaque assay. Of them, two showed significant activity (Table 2 and Table S2), namely MP-P5 (IC_50_ = 25.07 ± 0.05 µM, similar to activity against HIV [23]) and PP-P1 (IC_50_ = 1.08 ± 0.14 µM). Additionally, a treatment assay performed with MP-P5 and PP-P1 revealed that both PPCs efficiently inhibit ZIKV replication when added 1 h and 7 h post-infection (Figure S2). As observed for HIV [23], non-conjugated porphyrins did not show activity against ZIKV, reinforcing the claim that BBBpS conjugation is not only critical for BBB/BPB translocation but also for antiviral activity. On the other hand, and somewhat unexpectedly, PP-P1, shown to be inactive in vitro against HIV [23], emerged as the most active anti-ZIKV conjugate. As described in the literature, the light-independent mechanism of action of porphyrins is based on a direct perturbation of the viral envelope [25,31,32]. Porphyrins interact and accumulate on the envelope lipid membrane, causing a decrease in order and a consequent phase alteration that impairs viral entry processes. Since we ensured no-light conditions and no metal cations are coordinated to the porphyrin rings of the PPCs—avoiding generation of reactive oxygen species—the antiviral light-independent mechanism is the only plausible one. Thus, one may confidently suggest that the PP-P1 specific anti-ZIKV activity is guided by preferential interaction with the ZIKV viral envelope and/or components. 

### 3.4. Serum Stability 

**Figure 2:**
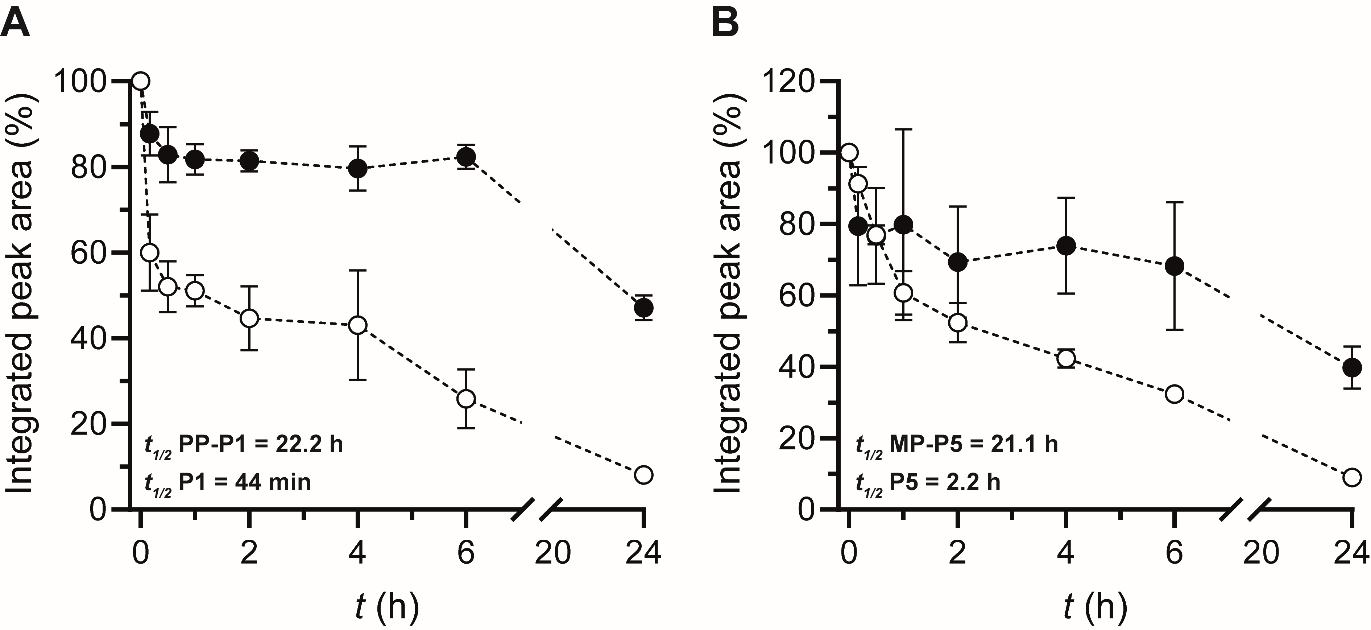
PPCs and peptides serum stability over 24 h. (**A**) PP-P1 (black) and P1 (white). (**B**) MP-P5 (black) and P5 (white).

The in vitro stability in human serum of all conjugates and their constitutive non-conjugated BBBpS was evaluated by LC and LC-MS as described [40,41]. PP-P1, the conjugate with highest ZIKV inhibitory activity, was remarkably resistant towards serum proteases (Figure 2A, black circles) with t_1/2_ > 22 h. This t_1/2_ is much higher than that of non-conjugated P1 (t_1/2_ = 44.0 min, Figure 2A, white circles), suggesting that the porphyrin payload in PP-P1 causes a steric shielding that occludes protease-sensitive sites in the P1 sequence. Comparably high t_1/2_ values were observed for P5-based conjugates (Figure 2B and Figure S3) which, given the clear differences in amino acid composition and/or charge (P1, cationic; P5, anionic), seem to exclude privileged features in the underlying BBBpS as the basis for preferential stability. Altogether, these data reinforce the view of the porphyrin ring somehow protecting cleavable peptide bonds from protease access. Interestingly, by comparing t_1/2_s, a general trend regarding the attachment site of the porphyrin ring can be discerned, with *N*-terminal conjugates showing higher stability (Figure S3A–D) than *C*-terminal ones (Figure S3E–H). In this last group, conjugation takes place through the side chain of an extra *C*-terminal Lys residue that the *N*-terminal series lacks. This Lys unit, however, cannot be viewed as contributing an additional cleavage point, since the ε-amino group on its side chain is involved in an amide bond with a porphyrin carboxyl and, hence, lacks the requisite positive charge for (trypsin-like) protease susceptibility.

An alternate explanation for the high t_1/2_s of some conjugates could invoke porphyrin moieties binding and/or blocking the machinery of a crucial set of serum proteases. While this view cannot be excluded outright, it seems less plausible because such an interaction would most likely affect indiscriminately most, if not all, conjugates, in contrast with the protease resistance observed exclusively in the P1 and P5 series (Figure S3). Finally, one may not exclude the aggregation behaviour of the different conjugates also playing a role in stability. In any event, further efforts (e.g., 3D NMR-derived structural models, zeta potential measurements, etc.) are clearly needed to establish either of the above hypotheses, but this exceeds the scope of the present work.

## 4. Conclusions

The potential of ZIKV to invade adult and foetus brains makes it one of the viruses of global concern. There are no effective drugs against ZIKV yet, mainly because developing molecules capable of overcoming the highly restrictive BPB and BBB to reach and inactivate the virus has proven extremely challenging. PPCs previously described by us [23] exhibited BBB translocation capacity in a mouse BBB in vitro model and promising activity against HIV and can, thus, be viewed as potential leads to fill the ZIKV treatment gap. Herein, we have addressed this issue by evaluating the in vitro BBB and BPB crossing ability and anti-ZIKV activity of eight new PPCs. We have identified PP-P1 as the most promising candidate, with elevated trans-BBB and -BPB scores and the highest antiviral potency. Moreover, PP-P1 has high serum stability, with a t_1/2_ > 22 h that bodes well for in vivo application. We do not have an explanation for the unique, manifold activity of PP-P1 among all PPCs examined, although one can conjecture various chemical, membrane and secondary cumulative interactions as a most likely scenario that deserves further in-depth studies. In any event, one may propose peptide–porphyrin conjugation as a promising strategy to tackle brain-resident viruses.

## Figures and Tables

**Table 1:** PPCs used in the study.

Abbreviation	Structure	[M + H^+^]^+^
MP-P1	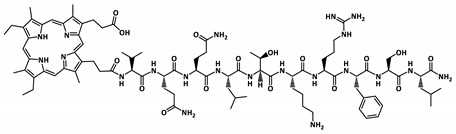	1767.0
PP-P1	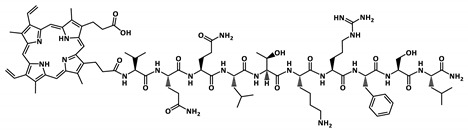	1762.9
P2-MP	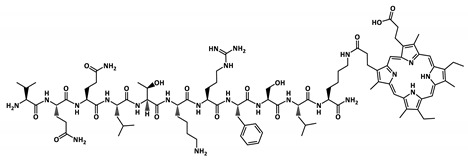	1895.1
P2-PP	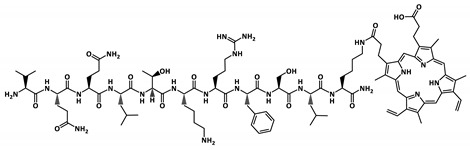	1891.1
MP-P5	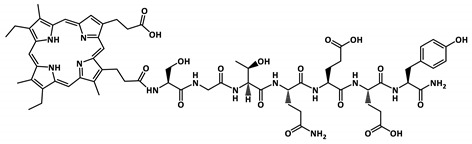	1360.6
PP-P5	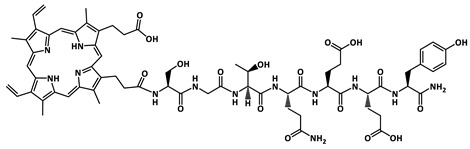	1356.6
P6-MP	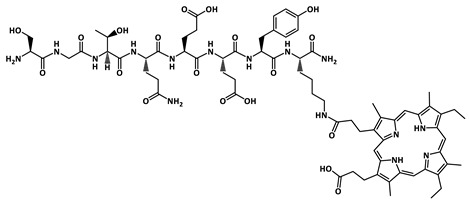	1488.7
P6-PP	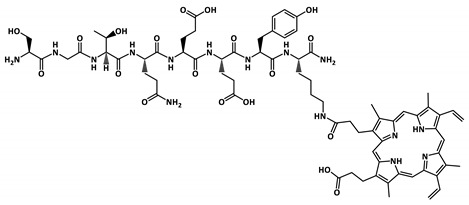	1484.7

**Table 2:** PPCs antiviral activity in vitro.

IC50 (µM)				
Virus	MP	PP	MP-P5	PP-P1
ZIKV	>50	>50	25.07 ± 0.05	1.08 ± 0.14
HIV ^a^	>50	>50	33.1 ± 1.38	>50

^a^ Values described at [24].

## Data Availability

Not applicable.

## Supplementary Materials

The following supporting information can be downloaded at: https://www.mdpi.com/article/10.3390/pharmaceutics14040738/s1, Table S1: Physicochemical properties of peptide shuttles used in the study; Figure S1: BPB in vitro model optimization. (A) Permeability of the model was evaluated based on the FD4-crossing throughout layers formed by the incubation of JEG-3 cells at densities ranging 1–4 × 10^4^ cells/cm^2^ in cell culture inserts. (B) Confocal microscopy imaging of BPB in vitro model [JEG-3 density of 1 × 10^4^ cells/cm^2^, ZO-1 (green) and Hoechst 33342 (blue)]. (C) Translocation capacity of antipyrine across the optimized BPB model (Translocation—grey, FD-4 permeability—black), Figure S2: Treatment of ZIKV-infected cells with PP-P1 and MP-P5. Vero cells were treated at 1 or 7 h post infection (h.p.i.) with 25 μM PP-P1 and MP-P5. After 24 h, the culture medium was collected and the released infectious virus particles were quantified by plaque assay as described in the Material and Methods of the main manuscript. Three independent replicates were performed, Figure S3: Serum stability of *N*-terminal conjugates (A, B, C, D) versus corresponding *C*-terminal conjugates (E, F, G, H). The conjugates labeling are as follows: A (MP-P1), B (PP-P1), C (MP-P5), D (PP-P5), E (P2-MP), F (P2-PP), G (P6-MP) and H (P6-PP), Table S1: Physicochemical properties of peptide shuttles used in the study, Table S2: PPCs global antiviral activity. HIV inhibition assays were performed as described at [2], Table S3: Final yield, HPLC purity and the mass of the synthesized PPCs, Scheme S1: Schematic representation of on-resin conjugation strategy at the *C*-terminus of the corresponding BBBpS.

## References

[1] Malone R.W., Homan J., Callahan M.V., Glasspool-Malone J., Damodaran L., Schneider A.D.B., Zimler R., Talton J., Cobb R.R., Ruzic I. (2016). Zika Virus: Medical Countermeasure Development Challenges. PLoS Negl. Trop. Dis..

[2] Chimelli L., Melo A.S.O., Avvad-Portari E., Wiley C.A., Camacho A.H.S., Lopes V.S., Machado H.N., Andrade C.V., Dock D.C.A., Moreira M.E. (2017). The spectrum of neuropathological changes associated with congenital Zika virus infection. Acta Neuropathol..

[3] Mlakar J., Korva M., Tul N., Popović M., Poljšak-Prijatelj M., Mraz J., Kolenc M., Resman Rus K., Vesnaver Vipotnik T., Fabjan Vodušek V. (2016). Zika Virus Associated with Microcephaly. N. Engl. J. Med..

[4] Parra B., Lizarazo J., Jiménez-Arango J.A., Zea-Vera A.F., González-Manrique G., Vargas J., Angarita J.A., Zuñiga G., Lopez-Gonzalez R., Beltran C.L. (2016). Guillain–Barré Syndrome Associated with Zika Virus Infection in Colombia. N. Engl. J. Med..

[5] Peiter P.C., Pereira R.D.S., Nunes Moreira M.C., Nascimento M., Tavares M.d.F.L., Franco V.d.C., Carvajal Cortês J.J., Campos D.d.S., Barcellos C. (2020). Zika epidemic and microcephaly in Brazil: Challenges for access to health care and promotion in three epidemic areas. PLoS ONE.

[6] Niemeyer B., Niemeyer R., Borges R., Marchiori E. (2017). Acute Disseminated Encephalomyelitis Following Zika Virus Infection. Eur. Neurol..

[7] WHO and Experts Prioritize Vaccines, Diagnostics and Innovative Vector Control Tools for Zika R&D. https://www.who.int/news/item/09-03-2016-who-and-experts-prioritize-vaccines-diagnostics-and-innovative-vector-control-tools-for-zika-r-d.

[8] Pattnaik A., Sahoo B.R., Pattnaik A.K. (2020). Current Status of Zika Virus Vaccines: Successes and Challenges. Vaccines.

[9] Waltz E. (2021). First genetically modified mosquitoes released in the United States. Nature.

[10] Albulescu I.C., Kovacikova K., Tas A., Snijder E.J., van Hemert M.J. (2017). Suramin inhibits Zika virus replication by interfering with virus attachment and release of infectious particles. Antivir. Res..

[11] Sacramento C.Q., De Melo G.R., De Freitas C.S., Rocha N., Hoelz L.V.B., Miranda M., Fintelman-Rodrigues N., Marttorelli A., Ferreira A.C., Barbosa-Lima G. (2017). The clinically approved antiviral drug sofosbuvir inhibits Zika virus replication. Sci. Rep..

[12] Demeule M., Regina A., Ché C., Poirier J., Nguyen T., Gabathuler R., Castaigne J.P., Béliveau R. (2008). Identification and design of peptides as a new drug delivery system for the brain. J. Pharmacol. Exp. Ther..

[13] Malakoutikhah M., Teixidó M., Giralt E. (2008). Toward an optimal blood-brain barrier shuttle by synthesis and evaluation of peptide libraries. J. Med. Chem..

[14] Malakoutikhah M., Guixer B., Arranz-Gibert P., Teixidó M., Giralt E. (2014). ‘À la Carte’ Peptide Shuttles: Tools to Increase Their Passage across the Blood-Brain Barrier. ChemMedChem.

[15] Oller-Salvia B., Sánchez-Navarro M., Giralt E., Teixidó M. (2016). Blood-brain barrier shuttle peptides: An emerging paradigm for brain delivery. Chem. Soc. Rev..

[16] Pardridge W.M. (1986). Receptor-mediated peptide transport through the blood-brain barrier. Endocr. Rev..

[17] Cavaco M., Frutos S., Oliete P., Valle J., Andreu D., Castanho M.A.R.B., Vila-Perelló M., Neves V. (2021). Conjugation of a Blood Brain Barrier Peptide Shuttle to an Fc Domain for Brain Delivery of Therapeutic Biomolecules. ACS Med. Chem. Lett..

[18] Zhang B., Sun X., Mei H., Wang Y., Liao Z., Chen J., Zhang Q., Hu Y., Pang Z., Jiang X. (2013). LDLR-mediated peptide-22-conjugated nanoparticles for dual-targeting therapy of brain glioma. Biomaterials.

[19] Banks W.A., Kastin A.J. (1985). Peptides and the blood-brain barrier: Lipophilicity as a predictor of permeability. Brain Res. Bull..

[20] Kumar P., Wu H., McBride J.L., Jung K.E., Hee Kim M., Davidson B.L., Kyung Lee S., Shankar P., Manjunath N. (2007). Transvascular delivery of small interfering RNA to the central nervous system. Nature.

[21] Neves V., Aires-Da-Silva F., Morais M., Gano L., Ribeiro E., Pinto A., Aguiar S., Gaspar D., Fernandes C., Correia J.D.G. (2017). Novel Peptides Derived from Dengue Virus Capsid Protein Translocate Reversibly the Blood-Brain Barrier through a Receptor-Free Mechanism. ACS Chem. Biol..

[22] Neves-Coelho S., Eleutério R.P., Enguita F.J., Neves V., Castanho M.A.R.B. (2017). A new noncanonical anionic peptide that translocates a cellular blood-brain barrier model. Molecules.

[23] Mendonça D.A., Bakker M., Cruz-Oliveira C., Neves V., Jiménez M.A., Defaus S., Cavaco M., Veiga A.S., Cadima-Couto I., Castanho M.A.R.B. (2021). Penetrating the Blood-Brain Barrier with New Peptide–Porphyrin Conjugates Having anti-HIV Activity. Bioconjug. Chem..

[24] Sakuma Y., Baba R., Arita K., Morimoto H., Fujita M. (2014). Food allergens are transferred intact across the rat blood-placental barrier in vivo. Med. Mol. Morphol..

[25] Neris R.L.S., Figueiredo C.M., Higa L.M., Araujo D.F., Carvalho C.A.M., Verçoza B.R.F., Silva M.O.L., Carneiro F.A., Tanuri A., Gomes A.M.O. (2018). Co-protoporphyrin IX and Sn-protoporphyrin IX inactivate Zika, Chikungunya and other arboviruses by targeting the viral envelope. Sci. Rep..

[26] Delcroix M., Riley L.W. (2010). Cell-penetrating peptides for antiviral drug development. Pharmaceuticals.

[27] Li S.Y., Cheng H., Qiu W.X., Liu L.H., Chen S., Hu Y., Xie B.R., Li B., Zhang X.Z. (2015). Protease-Activable Cell-Penetrating Peptide-Protoporphyrin Conjugate for Targeted Photodynamic Therapy in Vivo. ACS Appl. Mater. Interfaces.

[28] Dondi R., Yaghini E., Tewari K.M., Wang L., Giuntini F., Loizidou M., MacRobert A.J., Eggleston I.M. (2016). Flexible synthesis of cationic peptide-porphyrin derivatives for light-triggered drug delivery and photodynamic therapy. Org. Biomol. Chem..

[29] Lebedeva N.S., Gubarev Y.A., Koifman M.O., Koifman O.I. (2020). The Application of Porphyrins and Their Analogues for Inactivation of Viruses. Molecules.

[30] Biscaglia F., Gobbo M. (2018). Porphyrin–peptide conjugates in biomedical applications. Pept. Sci..

[31] Assunção-Miranda I., Cruz-Oliveira C., Neris R.L.S., Figueiredo C.M., Pereira L.P.S., Rodrigues D., Araujo D.F.F., Da Poian A.T., Bozza M.T. (2016). Inactivation of Dengue and Yellow Fever viruses by heme, cobalt-protoporphyrin IX and tin-protoporphyrin IX. J. Appl. Microbiol..

[32] Cruz-Oliveira C., Almeida A.F., Freire J.M., Caruso M.B., Morando M.A., Ferreira V.N.S., Assunção-Miranda I., Gomes A.M.O., Castanho M.A.R.B., Da Poian A.T. (2017). Mechanisms of Vesicular Stomatitis Virus Inactivation by Protoporphyrin IX, Zinc-Protoporphyrin IX, and Mesoporphyrin IX. Antimicrob. Agents Chemother..

[33] Eigenmann D.E., Jähne E.A., Smieško M., Hamburger M., Oufir M. (2016). Validation of an immortalized human (hBMEC) in vitro blood-brain barrier model. Anal. Bioanal. Chem..

[34] Jayant R., Atluri V., Agudelo M., Sagar V., Kaushik A., Nair M. (2015). Sustained-release nanoART formulation for the treatment of neuroAIDS. Int. J. Nanomed..

[35] Yin T., Yang L., Liu Y., Zhou X., Sun J., Liu J. (2015). Sialic acid (SA)-modified selenium nanoparticles coated with a high blood-brain barrier permeability peptide-B6 peptide for potential use in Alzheimer’s disease. Acta Biomater..

[36] Niewoehner J., Bohrmann B., Collin L., Urich E., Sade H., Maier P., Rueger P., Stracke J.O., Lau W., Tissot A.C. (2014). Increased Brain Penetration and Potency of a Therapeutic Antibody Using a Monovalent Molecular Shuttle. Neuron.

[37] Rothbauer M., Patel N., Gondola H., Siwetz M., Huppertz B., Ertl P. (2017). A comparative study of five physiological key parameters between four different human trophoblast-derived cell lines. Sci. Rep..

[38] Syme M.R., Paxton J.W., Keelan J.A. (2004). Drug transfer and metabolism by the human placenta. Clin. Pharmacokinet..

[39] Schneider H., Panigel M., Dancis J. (1972). Transfer across the perfused human placenta of antipyrine, sodium, and leucine. Am. J. Obstet. Gynecol..

[40] Cavaco M., Valle J., da Silva R., Correia J.D.G., Castanho M.A.R.B., Andreu D., Neves V. (2020). _D_ PepH3, an Improved Peptide Shuttle for Receptor-independent Transport Across the Blood-Brain Barrier. Curr. Pharm. Des..

[41] Gallo M., Moreno E., Defaus S., Ortega-Alvaro A., Gonzalez A., Robledo P., Cavaco M., Neves V., Castanho M.A.R.B., Casadó V. (2021). Orally Active Peptide Vector Allows Using Cannabis to Fight Pain while Avoiding Side Effects. J. Med. Chem..

