# Supplementary material for: Targeting Zika Virus with New Brain- and Placenta-Crossing Peptide–Porphyrin Conjugates
Source: Pharmaceutics. 2022 Mar 29;14(4):738. doi: 10.3390/pharmaceutics14040738 (PMC9032516; doi:10.3390/pharmaceutics14040738)
Supplement: Supplementary file 1 [file pharmaceutics-14-00738-s001.zip › pharmaceutics-1565900-Supplementary.pdf]

pharmaceutics

MDPI

Supplementary Materials: Targeting Zika Virus with New Brain- and Placenta-Crossing Peptide–Porphyrin Conjugates

Toni Todorovski, Diogo A. Mendonça, Lorena O. Fernandes-Siqueira, Christine Cruz-Oliveira [2], Giuseppina Guida, Javier Valle, Marco Cavaco, Fernanda I.V. Limas, Vera Neves, Íris Cadima-Couto, Sira Defaus, Ana Salomé Veiga, Andrea T. Da Poian, Miguel A.R.B. Castanho and David Andreu

Table S1. Physicochemical properties of peptide shuttles used in the study.

Entry number	Structure	$[M+H^+]^+$	Net charge at pH 7	Average hydrophilicity
P1	VQQLTKRFSL-amide	1218.7	3.00	−0.13
P2*	VQQLTKRFSL**K**-amide	1346.8	4.00	0.15
P5	SGTQEEY-amide	812.3	−1.00	0.54
P6*	SGTQEEY**K**-amide	940.4	0.00	0.85

*P2, and P6 are versions of P1 and P5, respectively, elongated with an extra Lys residue (in bold) to allow conjugation at the C-terminus.

BPB *In Vitro* Model Optimization

BPB *in vitro* model was optimized based on its permeability and monolayer formation. Firstly, JEG-3 cells were seeded in cell culture inserts, at densities ranging from 1 to 4 x 10^4 cells/cm^2, having their permeability evaluated, as described on the main manuscript, for 6 consecutive days (Figure S1A). The cells seeded at 1 x 10^4 cells/cm^2 display a slower and steadier decrease on its permeability during the 6 days, compared with the other densities tested, usually optimal for a single monolayer barrier formation. Secondly, the single monolayer formation was visualized by confocal microscopy. JEG-3 cells were seeded at a density of 1 x 10^4 cells/cm^2 in a μ-Slide 8 Well ibiTreat plate (Ibidi, Gräfelfing, Germany) and incubated for 6 days. After incubation, the monolayers were simultaneously incubated with ZO-1 antibody and Hoechst 33342 dye to stain tight junctions and the cell nucleus, respectively. Image acquisition was performed on a Zeiss LSM 880 confocal laser point-scanning inverted microscope (Carl Zeiss MicroImaging, Oberkochen, Germany) equipped with a C-Apochromat Corr 40x water immersion objective (1.20 numerical aperture). ZO-1 was excited using the Argon (488 nm) laser and Hoechst 33342 was excited using the Diode (405-30 nm) laser. For each experiment three different areas were imaged. All images were analyzed with the image processor Fiji. As observable at Figure S1B, the barrier formed is predominately constituted by a single monolayer, optimal for the BPB translocation assays. Lastly, the translocation capacity of antipyrine, a well described trans-BPB compound, across the establish monolayer was tested (Figure S1C), displaying values similar to the ones described in the literature [1].

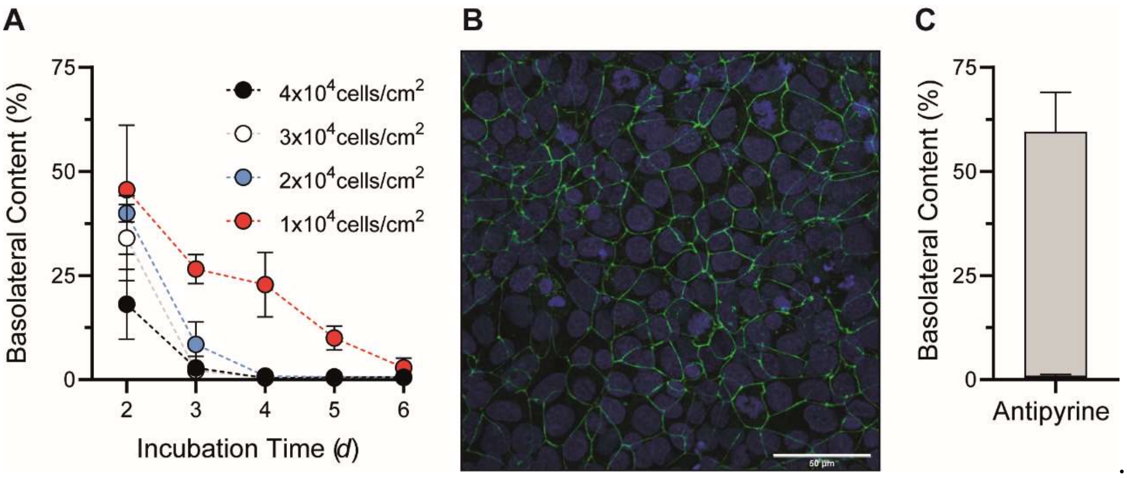

Figure S1. BPB in vitro model optimization. (**A**) Permeability of the model was evaluated based on the FD4-crossing throughout layers formed by the incubation of JEG-3 cells at densities ranging 1-4 x 10^4 cells/cm^2 in cell culture inserts. (**B**) Confocal microscopy imaging of BPB *in vitro* model [JEG-3 density of 1 × 10^4 cells/cm^2, ZO-1 (green) and Hoechst 33342 (blue)]. (**C**) Translocation capacity of antipyrine across the optimized BPB model (Translocation – grey, FD-4 permeability – black).

PPCs Global Antiviral Activity

Table 2. PPCs global antiviral activity. HIV inhibition assays were performed as described at [2].

	IC50 (μM)									
	**MP**	**PP**	**MP-P1**	**MP-P5**	**PP-P1**	**PP-P5**	**P2-MP**	**P6-MP**	**P2-PP**	P6-PP
ZIKV	> 50	> 50	> 50	25.07 ± 0.05	1.08 ± 0.14	> 50	> 50	> 50	> 50	> 50
HIV	> 50[a]	> 50[a]	> 50[a]	33.1 ± 1.38[a]	> 50[a]	3.02 ± 0.35	> 50[a]	> 50[a]	> 50[a]	5.28 ± 1.39

[a] values described at [2].

Treatment of Infected Cells with PCCs

Vero cells were infected with untreated ZIKV (1 MOI) as described in the Material and Methods of the main text, and then treated with PP-P1 and MP-P5 indicated times after infection. Virus released from the cells were quantified after 24 h.

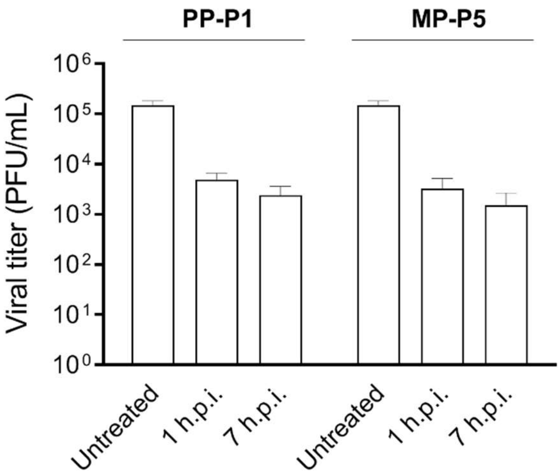

Figure S2. Treatment of ZIKV-infected cells with PP-P1 and MP-P5. Vero cells were treated at 1 or 7 hours post infection (h.p.i.) with 25 μM PP-P1 and MP-P5. After 24h, the culture medium was collected and the released infectious virus particles were quantified by plaque assay as described in the Material and Methods of the main manuscript. Three independent replicates were performed.

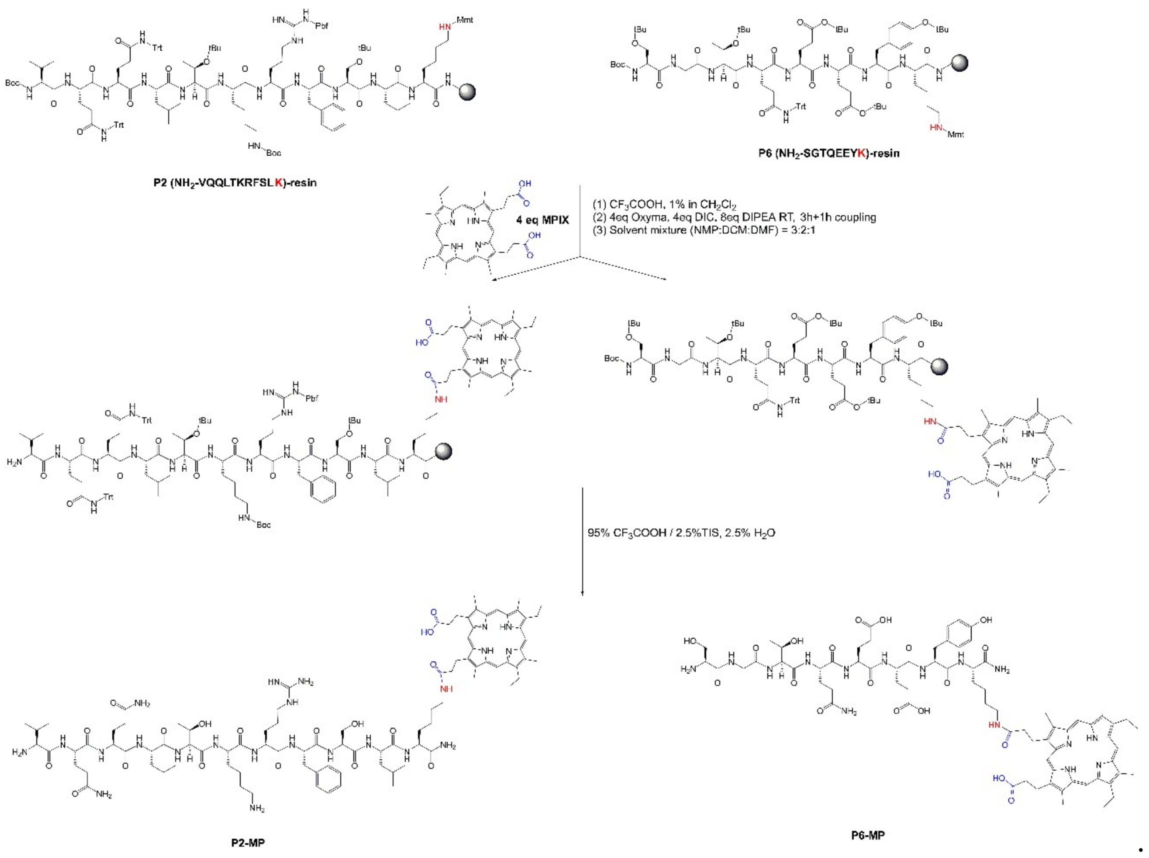

Scheme S1. Schematic representation of on-resin conjugation strategy at the *C*-terminus of the corresponding BBBpS.

Table S3. Final yield, HPLC purity and the mass of the synthesized PPCs.

Conjugate	Yield[a] (%)	Purity (%)	$[M+H^+]^+$
MP-P1	13.0	97.5	1767.0
PP-P1	14.8	93.8	1762.9
P2-MP	15.1	98.7	1896.1
P2-PP	14.2	92.4	1892.1
MP-P5	28.8	94.8	1360.6
PP-P5	8.0	92.0	1356.6
P6-MP	27.7	97.6	1488.7
P6-PP	14.3	91.1	1484.7

Prepared by the optimized protocol (see reference [2]).

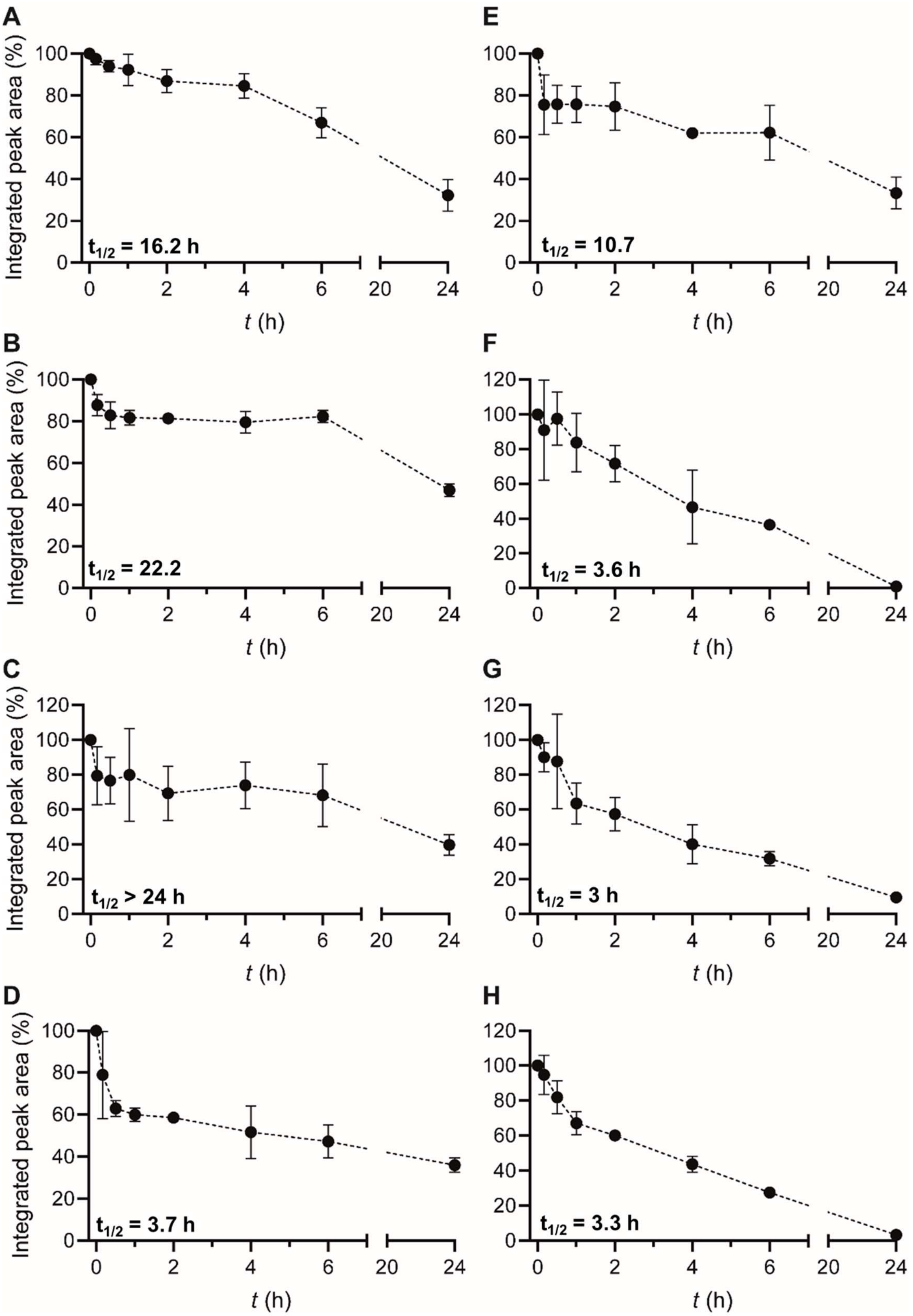

Figure S3. Serum stability of *N*-terminal conjugates (A, B, C, D) versus corresponding *C*-terminal conjugates (E, F, G, H). The conjugates labeling are as follows: A (MP-P1), B (PP-P1), C (MP-P5), D (PP-P5), E (P2-MP), F (P2-PP), G (P6-MP) and H (P6-PP).

REFERENCES

1. Schneider, H.; Panigel, M.; Dancis, J. Transfer across the perfused human placenta of antipyrine, sodium, and leucine. *Am. J. Obstet. Gynecol.* **1972**, *114*, 822–828, doi:10.1016/0002-9378(72)90909-X.

2. Mendonça, D.A.; Bakker, M.; Cruz-Oliveira, C.; Neves, V.; Jiménez, M.A.; Defaus, S.; Cavaco, M.; Veiga, A.S.; Cadima-Couto, I.; Castanho, M.A.R.B.; et al. Penetrating the Blood-Brain Barrier with New Peptide–Porphyrin Conjugates Having anti-HIV Activity. *Bioconjug. Chem.* **2021**, *32*, 1067–1077, doi:10.1021/acs.bioconjchem.1c00123.
